# Targeting environmental adaptation in the monocot model *Brachypodium distachyon*: a multi-faceted approach

**DOI:** 10.1186/1471-2164-15-801

**Published:** 2014-09-18

**Authors:** Matteo Dell’Acqua, Andrea Zuccolo, Metin Tuna, Luca Gianfranceschi, Mario Enrico Pè

**Affiliations:** Institute of Life Sciences, Scuola Superiore Sant’Anna, Pisa, Italy; Department of Field Crops, Namik Kemal University, Tekirdag, Turkey; Department of Biosciences, Università degli Studi di Milano, Milan, Italy

**Keywords:** Landscape genomics, *Brachypodium distachyon*, Adaptation, Genotyping by sequencing, Population genetics, GWAS, Association mapping

## Abstract

**Background:**

The local environment plays a major role in the spatial distribution of plant populations. Natural plant populations have an extremely poor displacing capacity, so their continued survival in a given environment depends on how well they adapt to local pedoclimatic conditions. Genomic tools can be used to identify adaptive traits at a DNA level and to further our understanding of evolutionary processes. Here we report the use of genotyping-by-sequencing on local groups of the sequenced monocot model species *Brachypodium distachyon*. Exploiting population genetics, landscape genomics and genome wide association studies, we evaluate *B. distachyon* role as a natural probe for identifying genomic loci involved in environmental adaptation.

**Results:**

*Brachypodium distachyon* individuals were sampled in nine locations with different ecologies and characterized with 16,697 SNPs. Variations in sequencing depth showed consistent patterns at 8,072 genomic bins, which were significantly enriched in transposable elements. We investigated the structuration and diversity of this collection, and exploited climatic data to identify loci with adaptive significance through i) two different approaches for genome wide association analyses considering climatic variation, ii) an outlier loci approach, and iii) a canonical correlation analysis on differentially sequenced bins. A linkage disequilibrium-corrected Bonferroni method was applied to filter associations. The two association methods jointly identified a set of 15 genes significantly related to environmental adaptation. The outlier loci approach revealed that 5.7% of the loci analysed were under selection. The canonical correlation analysis showed that the distribution of some differentially sequenced regions was associated to environmental variation.

**Conclusions:**

We show that the multi-faceted approach used here targeted different components of *B. distachyon* adaptive variation, and may lead to the discovery of genes related to environmental adaptation in natural populations. Its application to a model species with a fully sequenced genome is a modular strategy that enables the stratification of biological material and thus improves our knowledge of the functional loci determining adaptation in near-crop species. When coupled with population genetics and measures of genomic structuration, methods coming from genome wide association studies may lead to the exploitation of model species as natural probes to identify loci related to environmental adaptation.

**Electronic supplementary material:**

The online version of this article (doi:10.1186/1471-2164-15-801) contains supplementary material, which is available to authorized users.

## Background

One of the most ambitious objectives of natural variation studies is to provide a description of functional variability in natural populations [1]. The ability of a living organism to endure environmental challenges depends on the portion of genetic variation with adaptive implications [2] that sustains the formation of ecotypes through ecological evolution [3]. In plant sciences, being able to identify the genetic determinants of complex traits may help enhance crops [4]. The discovery of the genetic bases of complex traits with adaptive significance in model species [5] and in crops [6, 7] is often the first step towards molecular breeding programs [8, 9].

Domestication and breeding, however, have caused a severe reduction of crop diversity, whose extant genetic variation is much smaller than that of their wild relatives [10, 11]. This limits the diversity in which to search for adaptation, thus hindering our ability to identify favourable allelic combinations. Focusing on natural populations of the wild relatives of crops, with their broader genetic diversity, could help overcome this limitation and even allow new ground to be broken. As geographical objects, natural populations might be used to study the relation between the genetic and ecologic diversity in search of adaptive traits. Genomic synteny would then allow the targeting of homologous candidate adaptive genes in the crop of interest [12, 13]. The environment can be considered as an unceasing breeder selecting for successful alleles, providing this approach potential downfalls in an agronomic perspective.

The relation between genetic and climatic variation in natural populations has already been explored in humans [14, 15], and genetic determinants for fitness variation in different environments have been described in *Arabidopsis thaliana* [16]. Environmental data was gradually introduced in population genetics practises, being addressed by some landscape genetics and landscape genomics [17, 18], thereby being able to describe adaptive variability by means of the differential distribution of alleles on an ecological basis [19]. This can be done either through i) outlier detection or ii) association methods [20]. *Outlier detection* relies on Wright’s fixation index F_st_ [21] to identify loci under selection through their differentiation from the basal and neutral genomic variation [22]. Although widely used in animal species [23, 24] and less frequently in plant species [25], outlier detection can be biased by genetic structure and limited sensitivity [26]. In addition, it does not explicitly address environmental variation. On the other hand, *association* methods are based on marker - trait regressions and they directly target quantitative measures of the environment. The statistical framework of association methods is largely similar to that of genome-wide association studies (GWAS), which were originally developed in humans [27] to map complex trait determinants. GWAS are increasingly applied to plants [28, 29], where generally higher minor allele frequencies, multi-trait directional selection, and extensive linkage disequilibrium simplify their application [30].

When considering organisms with limited displacing abilities such as plants, association methods might accommodate quantitative environmental data as a response variable rather than phenotypes, and map genomic associations with climate [31–33]. Whilst outlier loci methods perform better with the strongest signatures of selection, association methods are appropriate to ascertain weak selection [26], and may lead to the identification of soft sweep signatures of low intensity selection [34, 35]. Outlier detection and association methods were merged in an investigation into *Populus* [36] and *Teosinte* [37], thus leading to the identification of loci with clear adaptive significance towards climate. A study in *Medicago* joined the association approach with an *ex situ* phenotypic evaluation, confirming the reliability of these methods [38]. In all cases, great focus is needed on to the interrelation of genetic variation and spatial displacement, as false statistical signals might arise when spatial structuration mirrors environmental adaptation [39]. The dependency of genetic diversity upon spatial diversity, though rarely considered in depth, can heavily influence the outcome of both these methods.

Merging population genomics and landscape data requires two sources of information. The *landscape* derives from geographical information systems (GISs), which can be used to couple quantitative geographical data with biological sampling [40, 41] and model the spatial relations of individuals. Global climate models developed for GISs [42] link climatic information with sampled individuals, providing both quantitative environmental data for each individual studied and a means for controlling spatial bias over genetic diversity. The *genomics*, in fact, must first consider the disturbance caused by the many evolutionary forces other than selection [43], as well as disturbance due to unknown demography that might add noise to association approaches [44]. High-throughput genotyping data are needed in order to provide the widest possible representation of the variation at a genome level, and thus efficiently control the many forces acting at such scale. The lowering of DNA sequencing costs together with the application of strategies for the reduction of genome complexity [45] makes DNA sequencing itself a means for discovering and analysing molecular markers [46]. Genotyping-by-sequencing (GBS) [47] is a reductionist strategy, and is increasingly employed in ecological genomics studies [48].

In this paper, we identify loci linked to environmental adaptation in Turkish accessions of the grass species *Brachypodium distachyon* (L.) P. Beauv. *Brachypodium distachyon* is the leading model species for small grain monocots and temperate grasses [49], with an ancestral range spanning the Middle and Near East, and currently including most of the temperate areas of the world [50]. Until recently, *B. distachyon* was deemed to have three distinct cytotypes of 2n = 10, 20 and 30 chromosomes: a recent study identified three different taxonomic entities, of which *B. distachyon* has the 2n = 10 chromosome set [51]. *B. distachyon* genome (approximately 271 Mbp) was completely sequenced in the inbred line Bd21 [52]. Natural populations of *B. distachyon* have already been extensively collected in Turkey, showing high intra-population homozygosity and a high level of inter-population genetic diversity [53]. This was an interesting condition to test the possibility to search for environmental adaptation whilst accounting for structuration.

We explored the possibility of identifying the relation between climate and genomic features in a starting panel of 82 *B. distachyon* individuals collected in nine locations scattered across a 1000-km transect in Turkey. By this, we wanted to exploit both methods developed in the landscape genomics field and in the GWAS community. Bringing landscape genomics closer to complex traits mapping, especially in an agronomical perspective, might open a significant perspective in the field. We employed a GBS approach to provide a genome-wide representation of molecular diversity in these *B. distachyon* individuals. The sampling locations were monitored on a GIS system to obtain climatic data for each individual, at the same time controlling for the spatial distribution of genetic diversity. The data was processed using the complementary characteristics of outlier and association approaches in order to identify signatures of adaptation at a molecular level.

We found that the association and outlier methods mostly targeted soft and hard sweeps of selection, respectively. GWAS and landscape genomics method jointly identified 15 genes involved in *B. distachyon* adaptation. We also found that transposable elements were differentially distributed across the genomes of local groups, some with a pattern matching the climatic diversity of the sampling transect.

Our method could be extended by including more genotypes and by targeting additional environments and environmental variables. Once the biological material is characterized, this might aggregate additional data and thus extend our capacity to understand the molecular bases of adaptation. *B. distachyon* could then be used as a *natural probe* to report functional variations in a broad set of environmental situations.

## Results

### GIS analyses and sampling

**Figure 1:**
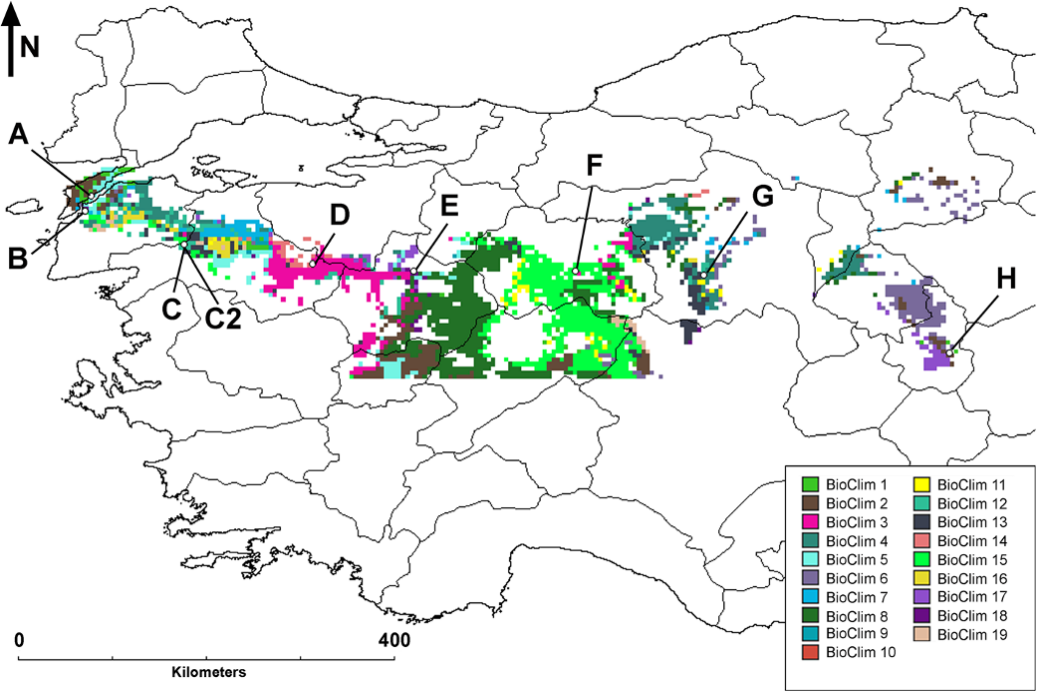
**GIS survey of the sampling area.** Depiction of the sampling transect as evaluated through DIVA GIS. False colours were generated to represent the most limiting factors for a typical annual grass species among the 19 BioClim variables through Ecocrop model. Letters A to H denote sampling locations and identify the 9 local groups analysed. Sampling locations were chosen to introduce the most possible environmental variation in relation to rainfall and temperature. BioClim variables are reported in legend. For full meaning, see Additional file 1. From west to east, altitude and aridity increase.

Nine Turkish *Brachypodium distachyon* local groups (Table 1) were sampled in separate locations in order to maximize environmental diversity. The map resulting from the Ecocrop modelling in DIVA-GIS (Figure 1) highlights the heterogeneous grid cells chosen for sampling. Geographical coordinates relative to the sampling locations were used to derive environmental data such as 19 BioClim variables and altitude. After normalization, environmental data was reduced by principal component analysis (PCA). The first three PCs accounted for 58.8%, 28.1% and 10.0% of the total variance. PC1 was positively correlated with altitude, temperature ranges, and negatively with rainfall. It represents the environmental gradient moving from western wet lowlands in Turkey to eastern dry uplands. PC2 was positively correlated with temperatures and weakly with altitude. PC3 was mainly correlated with isothermality, *i.e.* temperature evenness across the year (diurnal range over yearly temperature range) [Additional file 1].

**Table 1:** **Biological material included in the study**

Pop	Samples	Location	Longitude	Latitude
**A**	10	*Ilgardere-Gelibolu*	26.49027	40.27444
**B**	11	*18 Mart Üniv. Kampus*	26.43000	40.12527
**C**	10	*Yenice Balya arası*	27.39277	39.80000
**C2**	10	*Balya Yenice arası II*	27.41027	39.79111
**D**	10	*Dursunbey- Balıkesir*	28.64250	39.61416
**E**	10	*Kütahya Tavşanlı çıkışı*	29.63361	39.53944
**F**	10	*Kaymaz Mesire yeri Eskişehir*	31.20166	39.53888
**G**	10	*Polatlı- Haymana arası*	32.45583	39.50111
**H**	10	*Çanakkale Bursa Yolu Başlangıcı*	34.84166	38.74055

List of the natural populations of *B. distachyon* included in this study. At least 10 samples were chosen for each population. Pop codes A to H were given following a west–east transect across Turkey. Coordinates are given in WGS84.

### Genotyping by sequencing

The 96 samples were genotyped by sequencing, producing a total of 200,401,179 reads. The number of reads produced was rather uneven among the various individuals, thereby lowering the amount of usable polymorphic loci. The number of SNPs selected for GWAS (MAF >5%, call rate >80%) was 16,697. The comparison between the tested Bd21 inbred line and the Bd21 reference sequence produced only 148 polymorphisms (0.9%) of which 125 were due to heterozygous calls, thus supporting the correctness of SNP calling. The analysis of the distribution of reads showed that some genomic bins were consistently not sequenced in all samples sharing the same sampling area, whereas reads corresponding to the same bin were present in samples coming from other regions. 8,072 of such bins were characterized by either the presence or the absence of reads (P/A regions). Those regions were grouped into 4,911 continuous P/A regions spanning in length from the lowest arbitrary interval of 1,000 bp up to 22,000 bp (*x* = 1,819.2; *δ*^2^ = 1,779.5). 26.48% of the full genome of Bd21 was masked when scanned with a library of *B. distachyon* specific transposable elements (TE). The masking proportion rose to 45.96% in the 8,072 P/A, and dropped to 14.52% when an equal number of non-P/A regions randomly drawn from the genome were considered (Table 2).

**Table 2:** **Genomic distribution of transposable elements**

TE family	Whole genome	P/A	Non-P/A
**LTR-RT Ty1-copia**	4.86	5.06	1.51
**LTR-RT Ty3-gypsy**	13.63	29.76	8.19
**LTR-RT (tot)**	18.49	34.82	9.70
**DNA_TE**	5.41	5.87	3.30
**Other**	2.58	5.27	1.52
***Tot***	*26.48*	*45.96*	*14.52*

Enrichments of specific transposable elements repeats (TE family) in different collections of sequences; whole genome from Bd21, bins with presence/absence of reads (P/A), an equally dimensioned random set of bins without presence/absence patterns (non-P/A). LTR-RTs, the most common transposable elements family in plants, marks the biggest difference between P/A and non-P/A regions.

### Diversity analyses and population genetics

**Figure 2:**
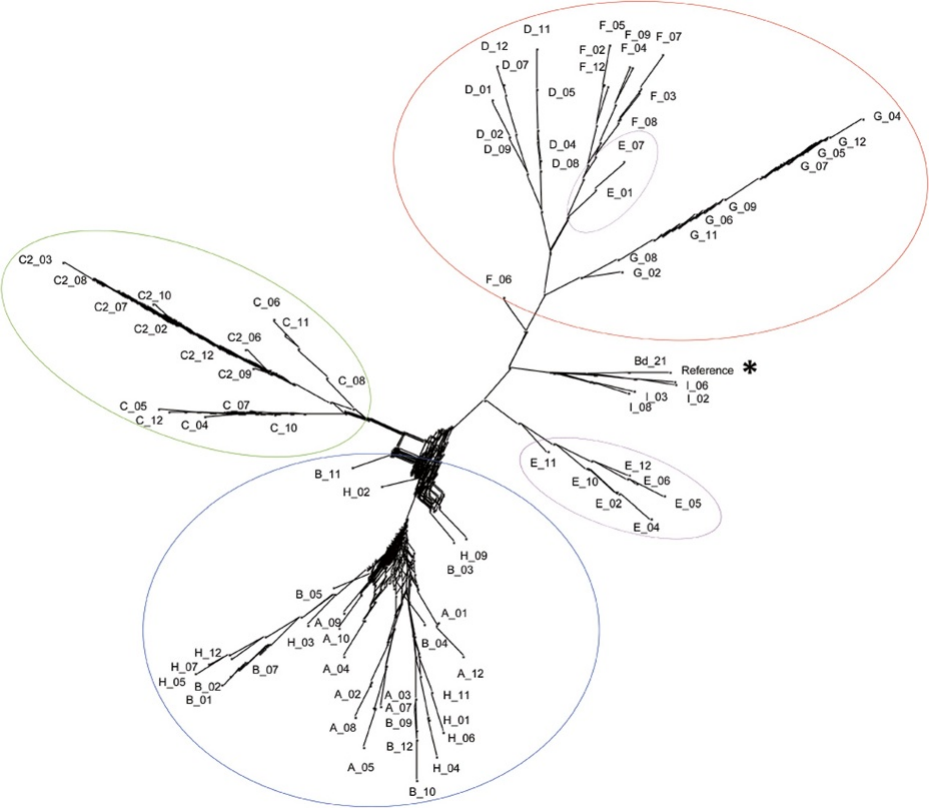
**Phylogeny based on the full set of SNPs.** Bootstrap network tree based on 1000 permutations with Uncorrected P distances. A-H correspond to the nine sampling locations listed in Table 1. All compatible splits are represented in a single branch; the more parallel branches there are, the more alternative splits were present in the bootstrapped dataset. The reference genome (Reference) overlaps with the Bd21 inbred line genotyped for control sakes (*), and clusters with the inbred lines (I). Local groups do not separate following a strict geographical criterion, yet within-group relationships are maintained. Circles encompass grouping of local groups A, B and H, local groups C and C2, and local groups D, F and G. Location E is intermediate, also geographically. The main split occurs between central Turkey groups and eastern and western sampling points.

The full set of filtered SNPs was used to produce a phylogeny by neighbour joining (NJ) clustering of uncorrected P distances, which highlighted an unexpected convergence in distant geographical areas (Figure 2). Overall, the analysed *B. distachyon* local groups were clustered into a few strongly supported clades. Individuals from the same sampling point mostly clustered together, suggesting a coincidence with biological populations having low variation. The local group E, split in two, was the sole exception. Interestingly, local groups did not cluster according to their spatial distribution. The westernmost (A, B) and easternmost (H) locales grouped with high confidence, in contrast to local groups D, F, G and partially E. Local groups C and C2, which were only 1.8 km apart, tightly clustered together but remained distinguishable, unveiling a low but detectable genetic differentiation at a small geographical scale.

**Figure 3:**
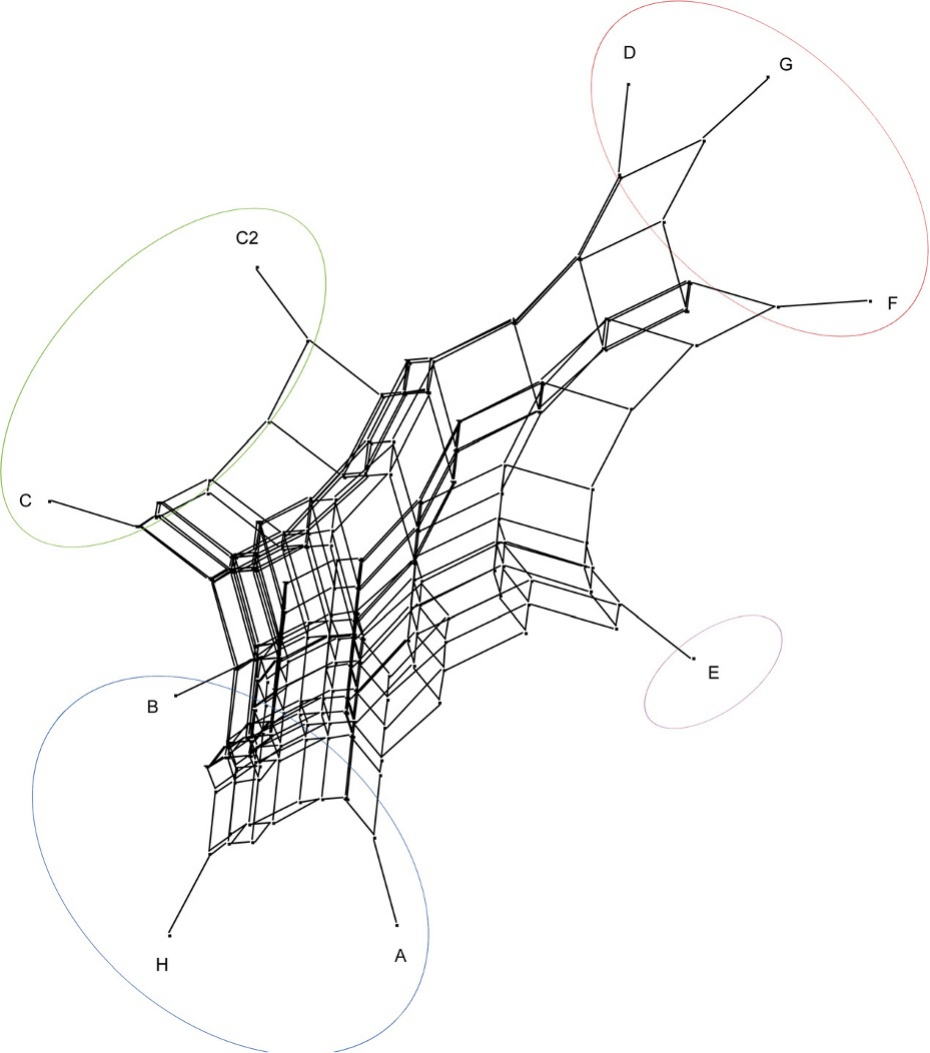
**Phylogeny based on P/A regions as group-wise markers.** A bootstrap network tree based on Jaccard’s distances of binary markers based on regions with consistent within-group presence/absence of reads. The tree topology, though more unstable, entirely overlaps with that produced by the SNPs in Figure 2. This suggests that distances deriving from P/A regions are primarily based on elements with segregation patterns similar to those of genetic variation, probably transposable elements and regions of DNA methylation.

The 8,072 P/A regions were converted into binary markers on a local basis and used to calculate distances between local groups with Jaccard’s similarity index. The resulting tree (Figure 3) is similar to that built from SNPs, suggesting that P/A regions are inherited in a similar way to molecular variation.

**Figure 4:**
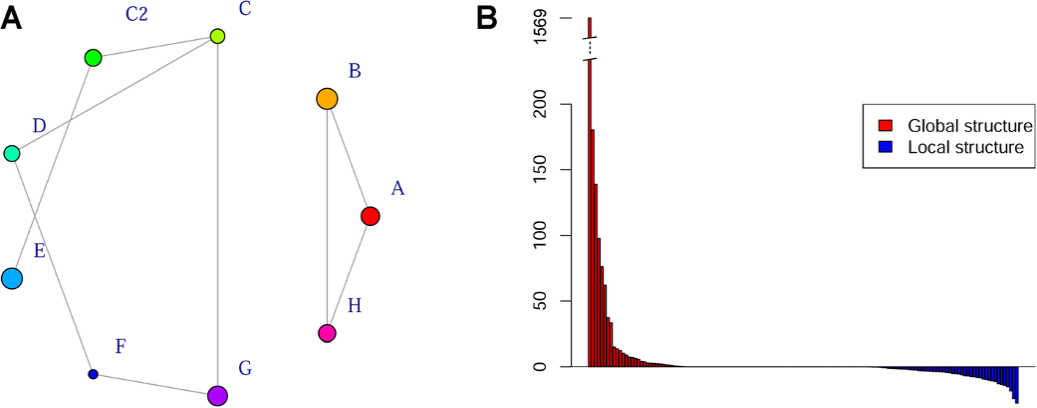
**Analysis of spatial structuration of molecular diversity.** The measure of conditional genetic distance is shown in panel **A**. Note that local groups are artificially set in a circle, so edge lengths are not proportional to conditional genetic distance. Node size is proportional to genetic diversity within sampling group. This graph confirms the detachment of sampling groups A, B, H from all of the others. In panel **B**, visual depiction of the spatial PCA. Positive PCs represent global structure, negative PCs local structure. Note the value of the first PC (out of scale). This global structure overlaps with the main split emerging from other analyses.

Pairwise F_st_ (Table 3) depicted a general scenario of scarce allele migrations and strong local fixation. When related to increasing geographical distances, however, the conditional genetic distance (cGD) showed almost no variation (Spearman *rho* = 0.1517 *pval* = 0.377). This does not mean that the panel is not spatially clustered. In fact, quite the opposite is true: gene flow is low if not absent (as confirmed by F_st_), especially between the two main clades also reported in the phylogeny. This is made clear by the incomplete population graph resulting from a spatial-aware molecular diversity analysis (Figure 4A). A spatial PCA also reported higher global than local genetic structure, and accounted most of the variance in the dataset to a single eigenvalue (Figure 4B). Again, this highlights the separation of sampling locations A, B and H from the rest.

**Table 3:** **Distance and diversity among populations**

Pop	A	B	C	C2	D	E	F	G	H
**A**		17.4	93.3	95.1	197.7	280.7	410.6	516.7	737.0
**B**	0.088		89.8	91.5	197.2	281.5	413.1	519.8	739.2
**C**	0.250	0.253		1.8	108.8	194.2	327.6	435.2	652.5
**C2**	0.465	0.458	0.565		107.2	192.6	326.1	433.7	650.9
**D**	0.674	0.680	0.744	0.845		85.7	220.0	327.7	543.8
**E**	0.213	0.212	0.280	0.434	0.573		134.6	242.4	458.3
**F**	0.598	0.603	0.664	0.767	0.360	0.484		107.8	326.6
**G**	0.708	0.713	0.788	0.901	0.614	0.609	0.418		222.7
**H**	0.098	0.068	0.258	0.448	0.671	0.222	0.599	0.700	

A to H, sampled populations from west to east. WGS84 coordinates in Table 2.

Population genetic parameters show that geographic distance does not influence population diversity. The lower matrix reports the estimated multilocus F_st_ among populations. The upper-right matrix indicates population pairwise distances in km.

A structure analysis conducted with Bayesian methods pointed to the existence of five distinct genetic clusters, thus extending the geographical pattern that had already emerged from the previous analyses. Samples from sampling locations A, B, C2, and H were all assigned to the largest cluster. D, F, G mostly accounted for the second largest cluster. All samples from region E but one clustered in a third cluster. Samples from region C shown some ancestry with those from C2 but acted as a separate cluster. The fifth cluster was contributed in small amounts by individuals from sampling location F. Overall, the spatial genetic diversity displays strong structuration, but little correlation with spatial distance.

### Genomic loci with adaptive significance

**Figure 5:**
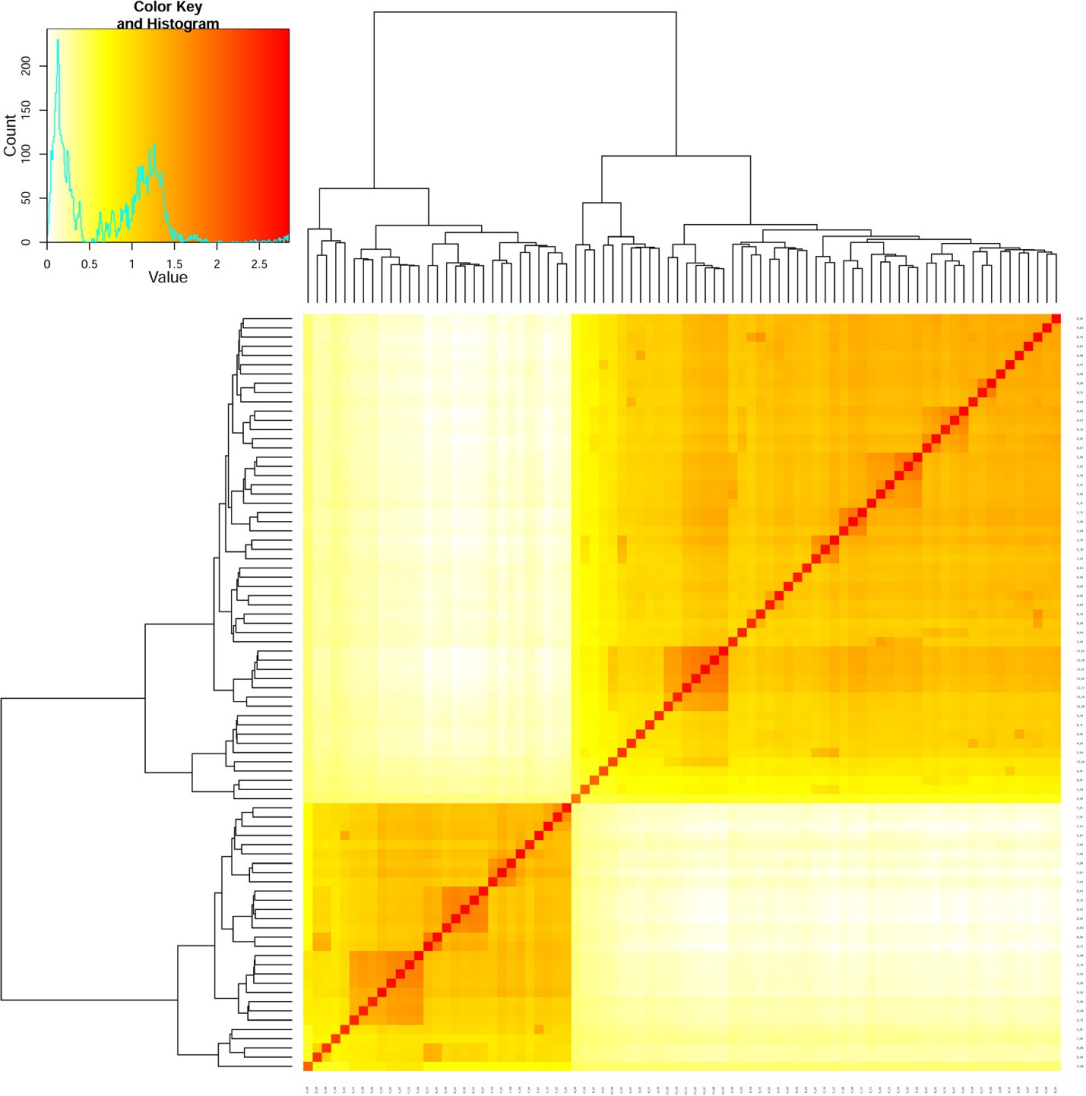
**Kinship analysis.** Kinship relationships among samples according to VanRaden method. Two main groups can be seen, the largest comprising mostly A, B, C, C2 and H individuals. The bimodal distribution of the kinship values confirm the results from diversity analyses, and probably would have biased the association analyses.

The genomics of adaptation were explored at three levels. The main approach was derived from association studies, using the first three PCs accounting for environmental data as a fixed variable. Latent factor mixed models (LFMM) were used to evaluate signals of environmental adaptation, controlling for false positives by considering the five cryptic genetic clusters identified by Structure. In parallel, a compressed mixed linear model (CMLM) considering kinship (K) and structure (Q) usually employed in GWAS analyses was also used to identify loci associated with climatic data. Kinship analysis confirmed the existence of two main genetic clusters already suggested by the previous analyses (Figure 5). When the cluster assignment provided by Bayesian clustering analysis was introduced as a covariate in the model, it over-corrected for structuration (data not shown). The PC method generally protects against structuration from genetic data [54–56] and was thus used together with kinship to correct the association analysis. The first five PCs calculated from molecular data were then used as Q by visually evaluating the normal fit of the quantile-quantile plots generated by the model [Additional file 2].

**Figure 6:**
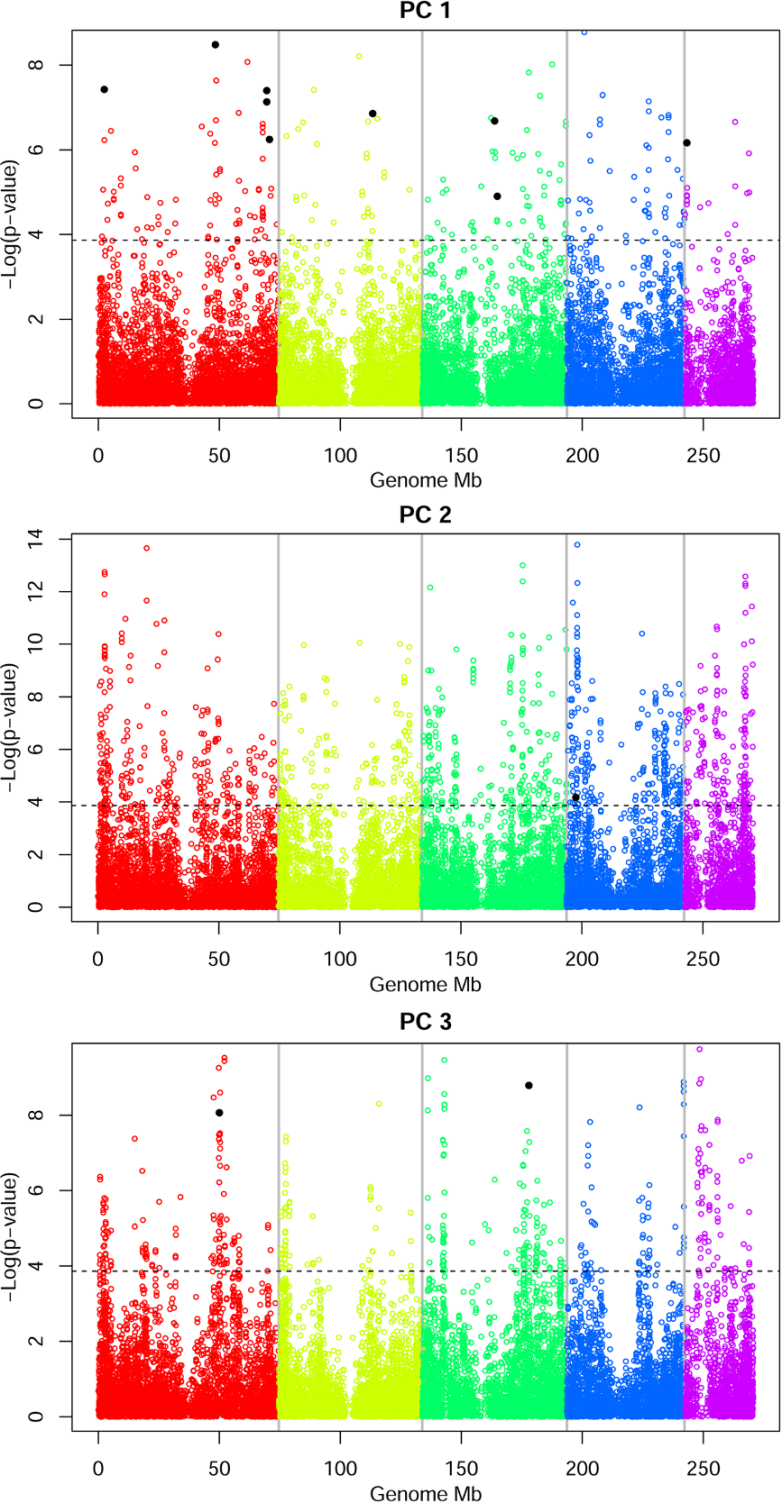
**Manhattan plots of the association tests.** Manhattan plots depicting association across the five *B. distachyon* chromosomes with environmental PCs 1 to 3, according to LFMM method. On the y axis, the significance of each association test; on the x axis, the SNP locations across the chromosome. The dashed line reports the significance for the LD-corrected Bonferroni method (*p-value* < 1.37 × 10^−4^). Black dots represent significant associations also detected with CMLM. The two methods identify clear peaks. Association peaks mostly have the skewed appearance given by linkage disequilibrium between cis elements nearby the strongest associations.

The outcome of both the association analyses was filtered with a corrected Bonferroni criteria accounting for the dependency of statistical tests within linkage blocks identified by a linkage disequilibrium (LD) analysis. The set of 82 *B. distachyon* samples chosen for association analysis showed a genome organized into 654 LD blocks containing between 2 and 492 SNPs. Eighty SNPs were not associated with any LD block. After using Bonferroni correction over 734 independent tests, every SNP that yielded a *p-value* lower than 1.37 × 10^−4^ (single test *p-value* < 0.1) with environmental PC variables was deemed to be an environment-associated SNP (EAS). The manhattan plot in Figure 6 merges the LFMM and CMLM output. Significant peaks have the expected skewed bell shape caused by linkage dragging markers nearby the most significant loci. Brachypodium.org was used to gather the corresponding protein domains from the Interpro database (http://www.ebi.ac.uk/interpro/), when available.

The LFMM approach identified alone 1035 genes, pointed by 901 genic EASs and 439 EASs in the 5 kb upstream predicted genes [Additional file 3] (note that a 5 kb upstream EAS may point to more than one gene). The CMLM was more conservative, reporting 18 genic EASs and 10 EASs 5 kb upstream predicted genes, identifying 30 predicted genes [Additional file 4]. When the two analyses were merged, this revealed 14 EASs pointing at 15 unique genes independently identified by both approaches (Table 4; [Additional file 3]).

**Table 4:** **Genes emerging from association analysis with climatic variables**

Chromosome	Position	ID	PC	Position
1	2493094	*Bradi1g02575*	1	genic
		*Bradi1g03700*	1	genic
1	69649973	*Bradi1g71690*	1	genic
1	69649974	*Bradi1g71690*	1	genic
1	70771534	*Bradi1g73170*	1	genic
2	38837316	*Bradi2g38560*	1	genic
3	29941038	*Bradi3g28560*	1	genic
3	44083155	*Bradi3g42530*	1,3	5 kb upstream
		*Bradi3g42540*	1,3	5 kb upstream
		*Bradi3g42550*	1,3	5 kb upstream
		*Bradi3g42560*	1,3	5 kb upstream
3	56650876	*Bradi3g56950*	1	genic
4	3872705	*Bradi4g04690*	2	5 kb upstream
		*Bradi4g04710*	2	5 kb upstream
5	1006046	*Bradi5g01110*	1	5 kb upstream
		*Bradi5g01120*	1	5 kb upstream

EASs confidently detected (*p-value* < 1.37 × 10^−4^) by both the association methods. The chromosome, EAS position in bp, gene ID and environmental variable involved (PC 1 to 3) are given. Each gene was identified by either an internal EAS (genic) or an EAS 5 kb upstream (5 kb upstream). Genes from *B. distachyon* annotation V1.2.

The aim of our second approach was to identify genomic loci under selection by applying a Bayesian outlier detection method. This analysis identified 953 outlier loci at an FDR of 0.05 (5.7% of the loci analysed). A total of 708 unique loci were either 5kbp upstream (247) and/or inside (461) 490 unique genes [Additional file 5]. Loci identified as outliers did not overlap with significant associations identified by CMLM. The other association approach, the least conservative LFMM, identified 75 SNP also being outlier loci, targeting 52 unique predicted genes highlighted in [Additional file 3]. The three methods showed an enrichment towards gene-related SNPs (Table 5).

**Table 5:** **Positional enrichment of SNPs identified by association and outlier methods**

Method	> 5 kbp upstream	< 5 kbp upstream	Genic	Total SNPs	Predicted genes involved
**LFMM**	276 (17%)	439 (27%)	901 (56%)	1616	1035
**GAPIT**	7 (20%)	10 (28.6%)	18 (51.4%)	35	30
**Bayescan**	245 (25.7%)	247 (25.9%)	461(48.4%)	953	490

Distribution of SNPs deemed significant in relation to predicted genes. Loci were grouped as outside predicted gene regions, within 5 kbp upstream of predicted genes, or within predicted genes.

**Figure 7:**
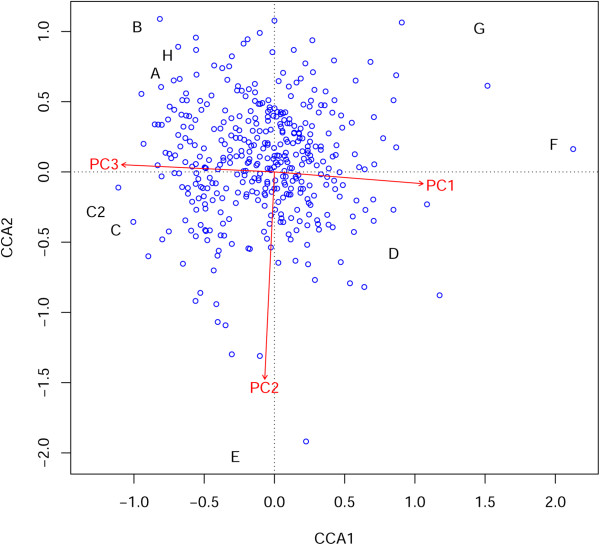
**Triplot from CCA.** The CCA analysis uses sites (sampling groups; letters) as fixed points to evaluate the relationship of individuals (P/A regions; dots), and environmental variation (PC1-3; vectors). Though many of the P/A regions are located near the centre of the graph, some appear highly related to PC1 and especially to PC2.

The third approach focused on the relation between P/A regions differentially distributed among *B. distachyon* locales and environmental PCs. A canonical correlation analysis (CCA) was used to quantify whether the environment could explain the differential distribution of P/A regions. The triplot in Figure 7 shows some of the P/A regions linearly related with environmental PC: this analysis can be read as a classical CCA in which sites are sampled groups (A to H), objects are P/A regions, and environmental vectors are represented by PCs. Constrained axes accounted together for 62.6% of the inertia. Sites/objects appeared linearly related to each of the three sites/variables at a *p* < 2.2×10^−16^ after 999 permutations.

### Putative genes involved in adaptation

We assayed the functional role of EASs detected by both association methods, as representatives of the strongest signal for adaptation (Table 4). Environmental PC1 targets most of the genic EASs. *Bradi1g03700*, a 60s ribosomal protein L36-3-like, is probably involved in expression control. In maize, the 60s subunit is involved in flooding responses [57] and might be related to environmental stress responses. The same EAS targets on the reverse strand *Bradi1g02575*, bearing an oxidoreductase activity domain. PC1 also targets a MYB transcription factor (*Bradi2g38560*) a class of proteins involved in plant responses processes, including those to abiotic stresses. MYB are a strategic targets for crop improvement [58]. Notably, we detected three outlier loci less than 100 Kb downstream this association [Additional file 3]. The phosphoprotein phosphatase *Bradi1g71690*, is likely involved in cellular signalling. Signalling is also contributed by *Bradi3g28560* (transferase activity). This predicted gene encodes for a 3-ketoacyl-CoA synthase, whose elective biological processes include wax synthesis [59] and response to cold and light stimulus (http://www.uniprot.org). The energetic balance of the cell is possibly contributed by *Bradi1g73170*, a sucrose transmembrane transporter targeted by PC1, and *Bradi4g04710*, targeted by PC2 and involved in the mitochondrial respiration chain. Within 500 Kb of this locus, two outlier loci are found [Additional file 3]. The EAS at 44,083,155 bp on chromosome 3, identified by both PC1 and PC3 is in the vicinity of a set of protein coding genes of unknown function.

## Discussion

### The twofold gain of genotyping by sequencing

Although genome re-sequencing offers the most inclusive possible overview of the genomic variability of small genome species [60, 61], methods based on the reduction of genome complexity such as GBS represent a cheaper and versatile alternative to genotype any species of interest in multiplex. However, due to the technical variations inherent in the protocol, a GBS run can yield an unbalanced representation of samples [62]. Here we showed that such an unbalanced distribution might mask biological reasons that are actually worth investigating.

In our case, the persistence of phylogenetic relationships among samples when using P/A regions as genetic markers (Figures 2 and 3) suggest that there is an inheritable pattern that is consistent with the differential distribution of transposable elements (TE) [63]. In this case P/A regions may either result from the loss of the cut site because of TE movement, or from the impairing of the methyl-sensitive ApeKI cleavage as a consequence of the presence of methylated DNA regions.

In both cases, the coverage of sequencing reads will show a gap as a result of the failure of the enzymatic cut. P/A regions are clearly enriched in TE, as demonstrated by the fact that the average TE content in those regions is significantly higher than that of the entire genome (45.96% versus 26.48%). The enrichment is even more dramatic when the TE content of regions not classified as P/A regions is taken into account (14.52%, more than three times less). This evidence strongly suggests that TE displacement has a role in the P/A polymorphism.

Two simple scenarios could be envisaged to explain the data: i) TEs inserted into the Bd21 reference genome after it separated from the other populations (or TEs inserted before Bd21 separated, but were then removed from some of the populations) thus giving rise to P/A polymorphism; ii) TEs are present in orthologous regions of both Bd21 and resequenced samples, but they are methylated only in some of the resequenced regions.

As far as we are aware this structural variation, as revealed by GBS, has never been reported before. We believe that it is of great importance as it may introduce a significant bias in genomic imputation. Our findings might stimulate further studies on the adaptive role of the differential distribution of transposable elements in *B. distachyon* natural populations. Our CCA analysis identified some of the P/A as being strongly related to environment, especially to PC1 and PC2 (Figure 7). Modifications in methylation patterns associated with transposable elements have already been reported to influence a set of genes in 20 maize lines [64].

### Approaching environmental associations

*Brachypodium distachyon* proved to be an effective model for the application of landscape genomics. The high F_st_ value between local groups (Table 3) is in accordance with the expectancy for self-fertilizing plants [65]. The depletion of intra-population variation in presence autogamy is exacerbated by selective sweeps, background selection, and possibly recurrent extinctions and recolonizations [66], as likely in our case. Our results might appear to be in contrast with those from *B. distachyon* populations from the Iberian peninsula, where SSR and ISSR markers showed an unexpectedly high intra-population variation [67]. We believe this might derive from the markers used, as SSR and ISSR sites change at a higher pace than coding regions targeted by GBS. In addition, as the authors suggest [67], the high variation in the Iberian populations might be linked to the proximity to the distribution limit of *B. distachyon.* On a broader scale, our SNP-based survey showed that the genetic diversity did not linearly correlate with spatial distances. As expected local groups are highly differentiated, yet share similarities with individuals far away (Figures 2, and 4). This is why the correlation between cGD and physical distance is not significant, but spatial structuration is both evident from the population graph and sPCA analyses (Figure 4).

Are we thus looking at isolation by distance (IBD)? IBD is the direct consequence of the limited dispersal of alleles, causing populations that are spatially near to share more similarities with each other than populations far away [68]. This phenomenon affects the exploitability of the molecular data derived from sampling natural populations [39]. The samples under study, though, do not show IBD in these terms. This is largely due to the split between locales A, B and H and all the others. While gene flow between local groups is low, there is no clear spatial pattern in the distribution of the genetic diversity.

Given the erratic nature of the sampling, we cannot rule out that patterns of gene flow between populations apply, at a finer scale, to IBD, as it is outside the scope of this work. However, the use of these results is a key feature for our association mapping approach. IBD, as in general spatial structuration, can mirror environmental association, leading to high rates of false positives. This was demonstrated in [39], where association methods without correction for population structure (such as SAM [69] and outlier loci discovery methods) found more significant associations than justified from the data if run in conditions of IBD. This happens in association methods because both climate and genetic variability have strong spatial dependencies which might lead to bias when overlapped. Hierarchical structure tests are also known to be possibly biased by IBD [39, 70]. Our analyses showed extensive non-linear spatial structuration, as expected since the autogamous reproduction of *B. distachyon*. This finding is in line with a previous survey performed with 43 SSR markers on 56 Turkish populations [53], where *B. distachyon* accessions split into two distinct phylogenetic clades differing in terms of vernalization habits and morphological features without belonging clearly to a specific geographical area.

However, the absence of a diversity gradient did not rule out structuration. We thus performed our association approach by considering structuration in order to avoid overrepresentation of false positives. This was done both with a hierarchical structure and a PCA with LFMM and CMLM, respectively, and we showed that the two different approaches yield similar results though differing in magnitude in terms of the statistical association found.

Our results are an empirical confirmation of what emerged in a simulation study testing the performance of five outlier-based and three correlation methods under explicit models for selection, demography and spatial relations [71]. In that study, the outlier detection implemented in Bayescan outperformed the other methods under any migration model, while all correlation-based methods proved powerful yet prone to bias due to structuration within and among populations. Nevertheless, if coupled with methods accounting for cryptic genomic structure, such methods could reduce type I and type II errors, especially in autogamous species. The portion of differentiated loci was in line with other studies [72], confirming that the use of a conservative FDR threshold (5%) and SNP filtering lowered the noise resulting from the use of a high number of polymorphisms.

In-gene polymorphisms are not the sole ones involved in environmental adaptation [38]. In fact, SNPs in genes and 5 kb window upstream of the genes (*i.e.* potentially involved in the regulation of gene expression) show an almost equal contribution to significant associations [73]. This also emerges from our association and outlier loci analyses, which revealed the EASs and outlier loci were enriched for genic and gene-related regions (Table 5).

### Lack of congruence between methods

An interesting point concerns the differences that emerged between outlier and association methods, which here report little loci in common. This result does not seem to fit the early tendency of seeing outlier loci as a confirmation for EAS validity and vice versa [69, 74]. Instead it highlights that association and outlier analyses estimate complementary aspects of functional adaptation, as recently suggested in similar studies [37].

The association approach is not dependent upon population genetic parameters, instead it targets a limited set of quantitative environmental characteristics. Complex traits targeted by means of correlative approaches, and especially those regarding climatic adaptation, are expected to reveal small changes in allele frequencies that push populations to a new optimum [35]. In this sense, polygenic selection [75] would seem to favour the simultaneous presence of multiple alleles rather than a complete fixation at the loci involved [34], resulting in the co-occurrence of different haplotypes at any given genomic location [76]. This contributes to the lack of congruence between the two methods, as a fainter signature of selection is less likely to be detected by outlier detection methods [77]. Unsurprisingly, a low intensity selection causes Bayescan to fail the most [26]. In addition, the LD-correction for false discovery rate possibly has an excessive number of type II errors [78]. However, these kinds of studies benefit from a more conservative threshold than from a permissive approach. A few loci are in fact expected to have high enough effects to be confidently detected.

Conversely, outlier methods do not depend, at least not directly, on environmental data. Loci identified by Bayescan but not by association methods might represent a set of loci under selection from factors not considered in the association analysis, such as fire regimes, soil composition, anthropic disturbance, grazing pressure, pathogens, and so on. Outlier methods are also affected by the assumptions about the null distribution used to compare loci, making the demographic history and structure of populations able to bias the outcome of the analysis [79, 80]. We argue that, at the net of false positives and negatives that might be effectively but not completely controlled by both methods, loci identified by both methods represent alternative portions of adaptive variation. Outliers represent the pool of loci under the strongest selection, whereas EASs represent the sum of the present and historical multilocus variations related to the environmental features considered.

A closer evaluation of the genes related to EASs identified by both the association methods provided a varied set of putative functions (Table 4). The annotation of *Brachypodium distachyon* is currently based mostly on *in silico* models, and therefore needs a careful evaluation of the functional relevance of EASs, which was outside the scopes of our experiments. Yet, we identified a set of genes, including a MYB transcription factor pointed by association and outlier loci, which already suggests the potential downstream applicability of these methods. Owing to the nature of LD, however, a less-than complete coverage sequencing cannot achieve the single-gene definition in association: our analyses revealed that the genome of our *B. distachyon* collection could be split into 734 LD blocks. To achieve a higher definition, more recombination events should be sampled, *i.e.* more individuals are needed. This is one of the strengths of this approach: since it is modular it allows the stratification of environmental and biological data in an integrated framework to map for adaptation in *B. distachyon*.

## Conclusions

We strongly support the application of next generation sequencing approaches to landscape genomics as a fast and modular tool for the discovery of adaptive traits, particularly in sequenced species. The application of landscape genomics to plants akin to crops can directly address adaptive variation that would be of great interest from an applied perspective. We noted that, when structuration is accounted for, the methodological effort to discover loci responsible for environmental adaptation might trace back to GWAS. This means that advances and statistics built by the complex trait mapping community could be exploited to gather information in the field.

Our results derive from a modular method that can be extended in order to deal with any relevant environmental questions. Although our initial set of genotypes and environmental variables is limited, we believe that this and similar collections will soon be enlarged to provide a better capacity to map environmental adaptation. *B. distachyon* - like other model species - is thus not only an effective laboratory tool, but also a natural probe. By exploiting their geographical distribution, these model species could be used to identify functional variation, and ultimately genomic loci, whose evolution was shaped for survival well before artificial selection took place. We envisage this approach being directly applied to crops, focusing either on their wild relatives or landraces, to cleverly incorporate in agronomy the results of natural selection efforts.

## Methods

### GIS analysis and sampling

The plant material studied comes from *B. distachyon* seeds collected in Turkey [53]. We focused on *B. distachyon* populations spanning from the western Dardanelles strait to the eastern region beyond lake Tuz in order to cover a continuous and comprehensive environmental gradient. This region was analysed by coupling DIVA GIS and BioClim data derived from Worldclim 2.5 minutes data (years ~1950-2000, ~5 Km) [81]. The function of the most limiting factor in Ecocrop model in DIVA GIS was used to identify a subset of locations maximizing climatic differences, reporting for each grid (5 × 5 Km) the BioClim variable with the lowest score with regard to general biological features for grasses.

A subset of nine local groups was chosen accordingly (Figure 1; Table 1). The sampling point C2 was chosen nearby C for control purposes. In each location, a minimum of 10 individuals were sampled as individual spikes bearing mature seeds. To ensure that the sampled individuals were reproducing (*i.e.* had non-zero fitness), we collected seeds rather than green tissues. Collection points were associated with GPS coordinates (±6 m), hence WGS84 coordinates were used to extract local altitude values and BioClim data from the Worldclim 2.5 database. BioClim is made up of 19 variables, the result of processing raw measures of rainfall and temperature. Using the full set of BioClim variables in correlation analyses might result in augmented noise without any real information gain [82], thus a PCA was conducted in R [83] over the 20 normalized environmental variables to extract the first three PCs.

### Genotyping

At least five seeds from each spike were pooled, and all sample pools underwent the same germination routine. Seeds representing each original individual were sown in separate Petri dishes with moist turf and underwent six weeks of vernalization in the dark. Seeds were then transferred to 1:1 turf and pebbly soil, and germinated in separated pots in a growth chamber (16 h 25°C light/ 8 h 21°C dark). Green tissues were collected in equal proportions from the resulting seedlings, so as to reconstitute the full allelic set of each original natural accession. Genomic DNA was extracted using the GeneElute Plant Genomic DNA Miniprep extraction kit (Sigma-Aldrich, St Louis, MO) following the suggested protocol. Four inbred lines developed by Dr. John Vogel in Albany, CA, USA, and the Bd21 inbred lines were added to the sample pool as reference. A total of 96 samples were selected for the following analyses.

The Genotyping-by-Sequencing (GBS) protocol is based on genome complexity reduction and multiplexed DNA sequencing for SNP discovery [47]. The protocol required a new adapter titration before being applied to *B. distachyon.* Total genomic DNA was digested with *Ape*KI restriction enzyme (120’ at 75°C; New England Biolabs, Ipswich, MA). Adapters were titrated by ligating Bd21 genomic fragments to increasing concentrations of adapters in separate reactions, then piping them through GBS library construction. After the library quality had been evaluated on a Bioanalyzer 2100 (Agilent Technologies, Palo Alto, CA), 6 ng of adapters per 100 ng of genomic DNA were deemed appropriate for all samples.

After adapter ligation with T4 ligase (New England Biolabs, Ipswich, MA) for 60’ at 22°C, then 30’ at 64°C, samples were pooled in two 48-plex cohorts and subjected to PCR amplification with high-fidelity Phusion DNA polymerase (New England Biolabs, Ipswich, MA) using adapter-specific primers. The two 48-plex libraries were treated following the Illumina pair-end sequencing protocol, and then sequenced in separate lanes on a Genome Analyzer II (Illumina, Inc., San Diego, CA) at IGA Services, Udine, Italy.

### Bioinformatics

An *ad hoc* script, available upon request, was used to carry out the following process on GBS Illumina reads: i) reads were sorted according to their barcode, ii) barcodes were removed from reads, iii) reads were trimmed according to their overall quality using the rNA program [84]. Trimmed reads were mapped onto the *B. distachyon* reference genome [52] using BWA software [85] run with the following settings: −n 3 -o 1 -e 1 -l 28, *i.e.* allowing three mismatches, disallowing long gaps, and using a seed length of 28 nucleotides. The results were analysed using the GATK pipeline [86]. GATK was used as it is the gold standard of SNP calls [87, 88]. At the time of the analyses Tassel software [89] was not capable of analysing paired-end sequencing data, and thus would have caused the loss of much information. The recommended identification and realignment of questionable aligned regions was carried out, and the actual SNP calls were made using the following settings: −stand_call_conf 50.0 -stand_emit_conf 10.0 -dcov 500 -out_mode EMIT_ALL_CONFIDENT_SITES. Alignments were edited and reformatted using SAM tools [90] and Picard tools (http://picard.sourceforge.net). Samples below the 9^th^ percentile of the distribution of read counts were discarded, thus reducing the number of individuals from 96 to 87, of which 82 were from field collection. Reads were mapped on the reference Bd21 genome sequence, and polymorphic positions were extracted.

The vcf files produced by GATK were parsed using a Perl script (available upon request): the analysis was limited to SNPs deemed as having PASSED by GATK (Phred-like quality score 50, *i.e. α<* 0.001%). All polymorphic positions missing in over 20% of the samples were discarded, and loci were filtered for minor allele frequency (MAF) of 5%.

The reference genome was split into arbitrary 1,000 bp bins, and the amount of reads mapped per bin per sample was counted to assess the consistency of the distribution of the reads. We labeled as Presence/Absence (P/A) regions those bins that were present in the reference genome but did not produce any read in any of the samples from one to eight of the groups (A-H) tested. In “absence” bins, no samples sharing the same geographical origin mapped any read, whilst one or more of the other groups did (with at least 1,000 sequenced reads per sample mapping on average). The content of transposable elements (TE) was assessed separately for P/A and non P/A regions using RepeatMasker [91] and a collection of *B. distachyon* TE as a repeat library (ftp://ftpmips.helmholtz-muenchen.de/plants/brachypodium).

### Diversity analyses

A phylogeny comprising both natural accessions and inbred lines was derived from shared SNPs. SplitsTree4 [92] was used to build a NJ phylogeny based on uncorrected P distances, and bootstrapping was used in 1000 replicates to build a bootstrap network based on all the alternative splits that had occurred [93]. The degree of kinship among individuals was estimated from molecular data in R/GAPIT [94] using VanRaden's [95] method. P/A regions were used to derive binary markers (1/0) to mark the presence or absence of sequences in each genomic bin in each local group, and a distance matrix was calculated on the basis of Jaccard distances, hence considering shared states only. This method does not require any assumption on the biological nature of P/A regions.

Gene flow dynamics underlying the geographical sampling can affect the results of the analyses, and need to be considered in landscape genomics practises [39, 71]. Genepop 4.1.4 [85] was used to estimate Wright’s fixation index (F_st_) [31]. The genetic distance among local groups was measured as the conditional genetic distance (cGD) [96], a measure derived from population graphs [97], which by accounting for spatial variance outperformed classical measures of genetic distance [96, 98]. In a population graph each population or group of individuals is identified by a node on a graph, and nodes are connected by edges whose length (cGD) is inversely related to the genetic covariance between populations. Null length, *i.e.* unconnected nodes, represent populations lacking allelelic exchange. cGD values were regressed over spatial distances.

The spatial pattern of genetic diversity was explored at a finer scale with a spatial PCA [99] in R/adegenet [100]. This method summarizes both the spatial structure and the genetic diversity among individuals, thus enabling global and local spatial structures to be differentiated. Structure [101] was used in admixture model to survey the number of cryptic genetic clusters (K) present in the dataset. The most likely K was identified by structure harvester [102].

### Landscape genomics

Association analysis was performed with two different methods on the full set of SNPs filtered for MAF > 0.05 against the three PCs accounting for environmental variation. LFMM software [103] was used to exploit latent factor mixed models over the full set of SNPs. This method is aimed at controlling population history and IBD to control type I errors in gene-environment associations. This is done by considering genetic structures (K) as unobserved variables. We ran the analysis iterating K from 1 to 10, three replicates each, for each of the environmental PC axes. After observing the outputs of the model, we chose K according to the number of clusters detected by Structure. LFMM was run with 1,000 burning sweeps and 10,000 effective sweeps. The other method to association mapping uses R/GAPIT [94]. This represents a proper GWAS association approach, built onto multiple *F-tests* between a full model against a reduced model at each marker. R/GAPIT enables a compressed mixed linear model (CMLM) [104] to deal with any data potentially perturbed by population structure and kinship. This approach reduces type I (while possibly increasing type II) errors [105] and can be described as in [94]:


Where *Y* is the vector of phenotypic/climate values, and *X* and *Z* are the known design matrix. The fixed effects (genetic marker, intercept and population structure (Q)) are represented by the unknown vector *β*; random additive genetic effects are represented by the unknown vector *u*, while *e* represents the non-observed residuals. Kinship is included in the computation of *u* and *e* variance. The most significant PCs computed over molecular markers and the Structure clustering were evaluated as Q by assessing the normal fit of the model on quantile-quantile plots.

To control for false positives we applied an LD-corrected Bonferroni. The Bonferroni method is conservative in that it divides the target threshold (*e.g.* 0.05) by the number of tests performed. However GWAS is not necessarily a collection of completely independent tests [78, 106]. This is because the genetic and functional linkage among markers, expressed by LD, causes SNPs to be inherited in linkage blocks rather than independently. This is especially true in natural populations of autogamous plants with extensive LD [107]. R/trio [108] was used to compute pairwise LD in 500 marker windows (8 Mbp on average). The normalized *D’* LD measure was used to identify LD blocks where strong LD was defined by an upper confidence bound of *D’* > 0.98 and a lower confidence bound of *D’* > 0.7. Strong evidence of recombination was provided wherever the upper bound of *D’* was lower than 0.9, according to Gabriel's method [109]. We established a threshold corresponding to one false association out of ten (0.1) and divided it by the number of linkage blocks in order to have LD-corrected Bonferroni FDR.

The same dataset was tested to detect outlier loci (*i.e.* loci under selection) using Bayescan 2.1 [110]. This method entails decomposing F_st_ values in a locus-specific component (α; shared by all populations), and a population-specific component (β; shared by all loci). The departure of α from the equilibrium suggests selection operating on a given locus. The 5% FDR threshold provided by Bayescan was used as a significance threshold. *Brachypodium distachyon* genome V1.2 annotation (ftp://ftpmips.helmholtz-muenchen.de/plants/brachypodium/v1.2) was used to locate EASs and outliers either more than 5 kb upstream, within 5 kb upstream, and within predicted genes with R/GenomicRanges [111]. The limit of 5 kbp was chosen as being representative of possible *cis* regulatory regions [73]. To avoid redundancy, SNPs falling at the same time into a predicted genic region and 5 kb upstream of another predicted genic region, were considered once and genic only. The list of outliers was compared with that of the EASs significant for either of the two association methods. SNPs identified by at least two methods were further discussed as strong adaptation candidates.

P/A regions as binary markers were used in a canonical correspondence analysis (CCA) [112] with R/vegan [113]. A CCA is used in ecological studies to evaluate the amount of variability of a matrix of observations X is explained by a matrix of descriptive variables Y referring to the same sites where observations are made. Typically, CCA is used to assess the unconstrained relation between environmental factors and species distribution, but can also be used to associate climate gradients with molecular data [114]. We used CCA to evaluate the linear relation existing between P/A regions and environmental PC with 999 permutations.

## Supporting data

All sequencing reads from this study have been submitted to the European Nucleotide Archive (http://www.ebi.ac.uk/ena/) under accession no. PRJEB7130. Biological materials are available upon request. Climate data is publicly available at http://www.worldclim.org.

## Electronic supplementary material

Additional file 1: **Original meaning of BioClim variables and correlation with the first three PC axes.** The bar plot shows the correlation between altitude, the 19 BioClim variables and environmental PC 1 to 3 (numerical data reported below). Aside, the original meaning of BioClim variables. (XLSX 18 KB)

Additional file 2: **Association analysis quantile-quantile plots for environmental PC 1–3.** Quantile-quantile plots generated by GAPIT model for PC 1 to 3. On the y axis, the distribution of calculated p-values. On the x axis, the expected distribution of association test statistics. A few, strong associations are present. (PDF 1 MB)

Additional file 3: **Genes detected by LFMM association method.** Predicted genes identified by significant associations detected by LFMM approach. Entries also identified by outlier discovery approach are underlined and bold; entries also identified by CMLM association are highlighted in red. The three methods show interesting overlaps (discussed in text). (XLSX 82 KB)

Additional file 4: **Genes detected by CMLM association method.** Predicted genes identified by significant associations detected by CMLM approach, with relative physical position and test significance. (XLSX 27 KB)

Additional file 5: **Genes detected by outlier loci analyses.** List of predicted genes identified by outlier loci approach, with relative physical position and test significance. (XLSX 59 KB)
