# Supplementary figures and images for: Targeting environmental adaptation in the monocot model Brachypodium distachyon: a multi-faceted approach
Source: BMC Genomics. 2014 Sep 18;15:801. doi: 10.1186/1471-2164-15-801 (PMC4177692; doi:10.1186/1471-2164-15-801)

**.PCA1**

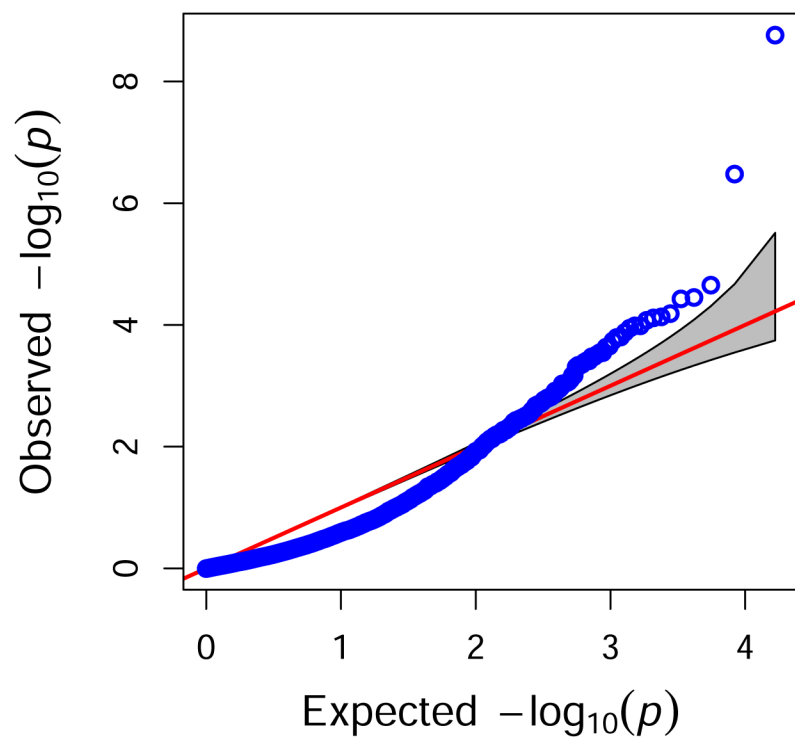

**.PCA2**

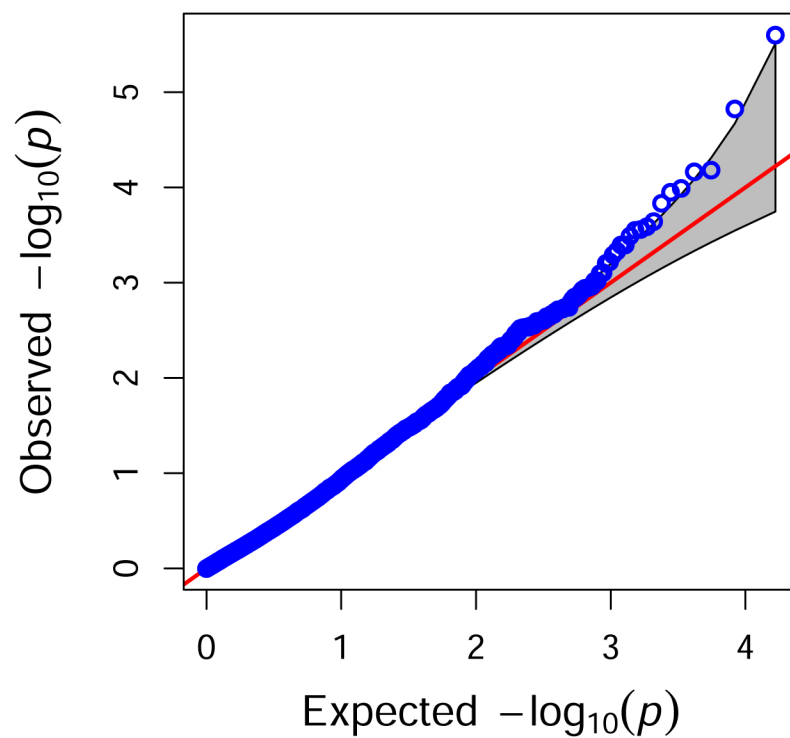

**.PCA3**

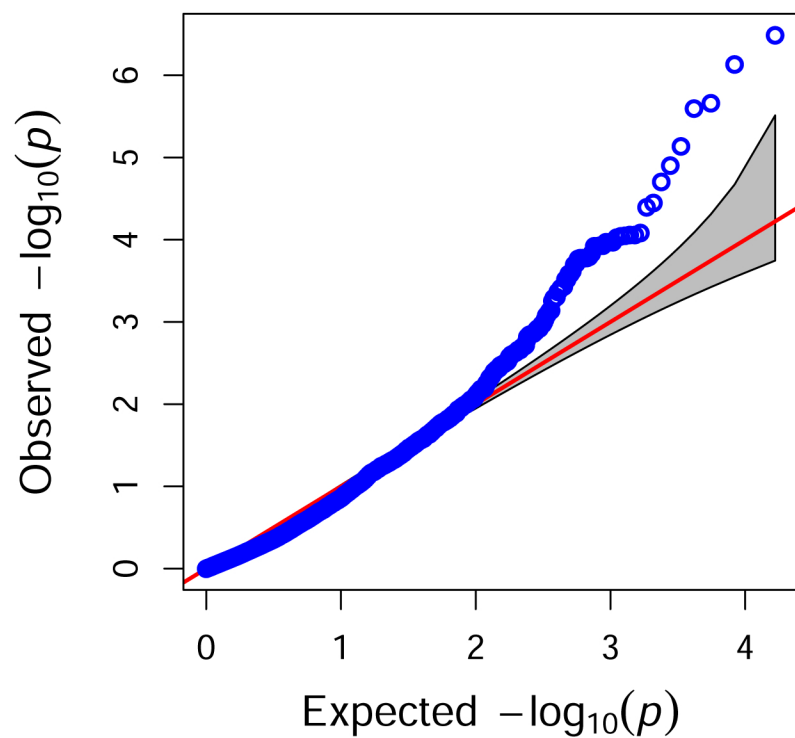

Supplement: Supplementary file 2 — Additional file 2: Association analysis quantile-quantile plots for environmental PC 1–3. Quantile-quantile plots generated by GAPIT model for PC 1 to 3. On the y axis, the distribution of calculated p-values. On the x axis, the expected distribution of association test statistics. A few, strong associations are present. (PDF 1 MB) [file 12864_2014_6482_MOESM2_ESM.pdf]
